# Supplementary material for: Stochastic gene expression and environmental stressors trigger variable somite segmentation phenotypes
Source: Nat Commun. 2023 Oct 14;14:6497. doi: 10.1038/s41467-023-42220-7 (PMC10576776; doi:10.1038/s41467-023-42220-7)
Supplement: Supplementary file 1 — Supplementary Information [file 41467_2023_42220_MOESM1_ESM.pdf]

Supplementary Information

Stochastic gene expression and environmental stressors trigger variable somite segmentation phenotypes

Kemal Keseroglu[1], Oriana Q.H. Zinani[1,2], Sevdenur Keskin[3], Hannah Seawall[1], Eslim E. Alpay[1], and Ertuğrul M. Özbudak[1,4,*]

[1]Division of Developmental Biology, Cincinnati Children's Hospital Medical Center, Cincinnati, OH, 45229.

[2]Molecular and Developmental Biology Graduate Program, University of Cincinnati, College of Medicine, Cincinnati, OH, 45229.

[3]Department of Allergy and Immunology, Albany Medical College, Albany, NY, 12208.

[4]Department of Pediatrics, University of Cincinnati College of Medicine, Cincinnati, OH, 45229.

*Correspondence: ertugrul.ozbudak@cchmc.org

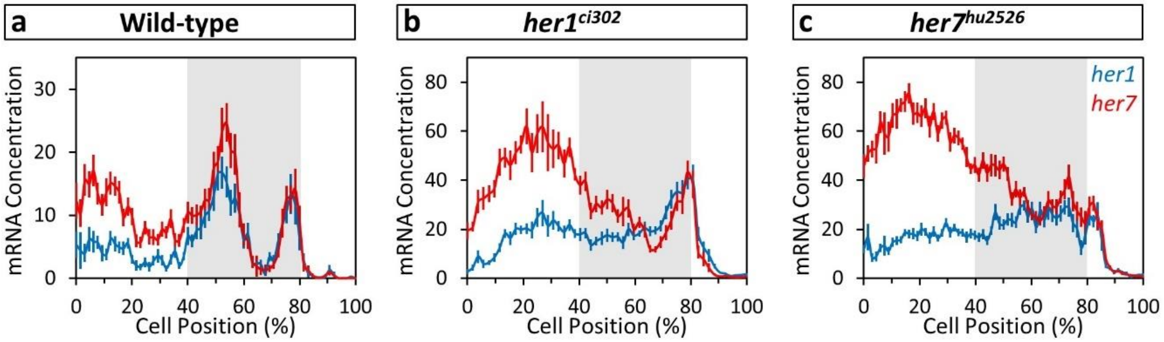

Supplementary Figure 1. *her1* and *her7* have a high transcriptional correlation in wild-type embryos compared to *her1*^{ci302} and *her7*^{hu2526} mutant embryos. a-c Profile of mean *her1* (blue) and *her7* (red) RNAs in a wild-type (**a**), a *her1*^{ci302} (**b**), and a *her7*^{hu2526} (**c**) mutant embryos along the posterior-to-anterior PSM. Gray box shows 40-80% of PSM. Error bars indicate two S.E.M.

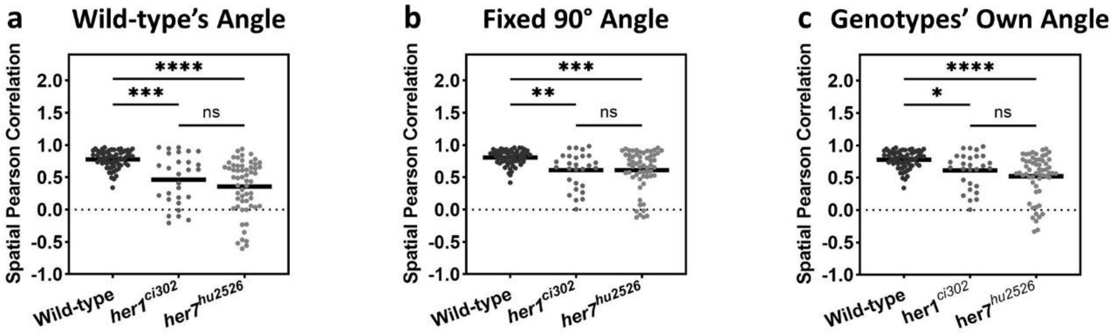

Supplementary Figure 2. Slicing the PSM with different angles shows similar spatial Pearson correlations. a-c The spatial Pearson correlation scores of *her1* and *her7* using wild-type's angle (**a**), fixed 90° angle (**b**), and genotypes' own angle (**c**), which is seen in Fig 2d. ***P = 0.0005 (wild-type versus *her1*^{ci302}), ****P = 0.2175x10^{-9} (wild-type versus *her7*^{hu2526}), P = 0.4385 (*her1*^{ci302} versus *her7*^{hu2526}) in (**a**), **P = 0.0029 (wild-type versus *her1*^{ci302}), ***P = 0.0006 (wild-type versus *her7*^{hu2526}), P > 0.9999x10^{-11} (*her1*^{ci302} versus *her7*^{hu2526}) in (**b**), and *P = 0.0157 (wild-type versus *her1*^{ci302}), ****P = 0.2146x10^{-5} (wild-type versus *her7*^{hu2526}), P = 0.7243

(*her1*^{ci302} versus *her7*^{hu2526}) in (**c**), Kruskal–Wallis ANOVA with Dunn's multiple-comparison correction. ns, not significant. Black lines show the mean of data in the scatter dot plot.

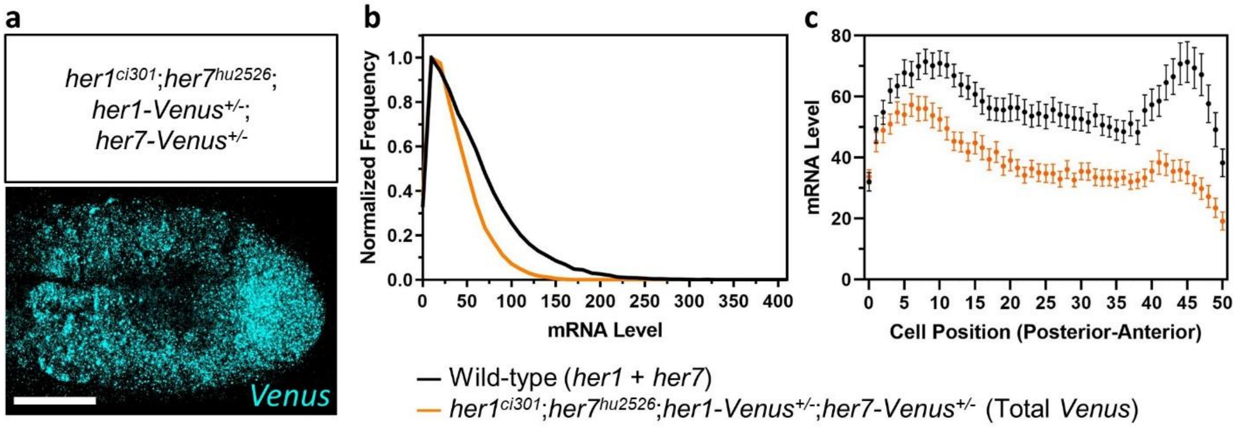

Supplementary Figure 3. Live reporters are partially functional. a smFISH image shows total *Venus* expression (cyan) along the PSM of *her1*^{ci301};*her7*^{hu2526};*her1-Venus*$^{+/-}$;*her7-Venus*$^{+/-}$ embryos. Scale bar is 100 μm. **b** The frequency histogram of total *her* (*her1*+*her7*, black) RNA per cell in wild-type (mean=55, *n*=31, *N*=3) and total *Venus* (*her1-Venus*+*her7-Venus*, orange) RNA per cell in *her1*^{ci301};*her7*^{hu2526};*her1-Venus*$^{+/-}$;*her7-Venus*$^{+/-}$ embryos (mean=35, *n*=18, *N*=2). The mean of transgene RNAs in mutant embryos was 36% lower than the mean of endogenous (*her1* plus *her7*) RNAs in wild-type embryos. **c** The mean of transgene RNAs (averaged from posterior to anterior PSM) is 33% lower than that of the endogenous RNAs in wild-type embryos. The anterior end of the PSM length is truncated to match the PSM length of the shortest samples. Error bars indicate S.E.M. *n* is the number of embryos; *N* is the number of independent experiments.

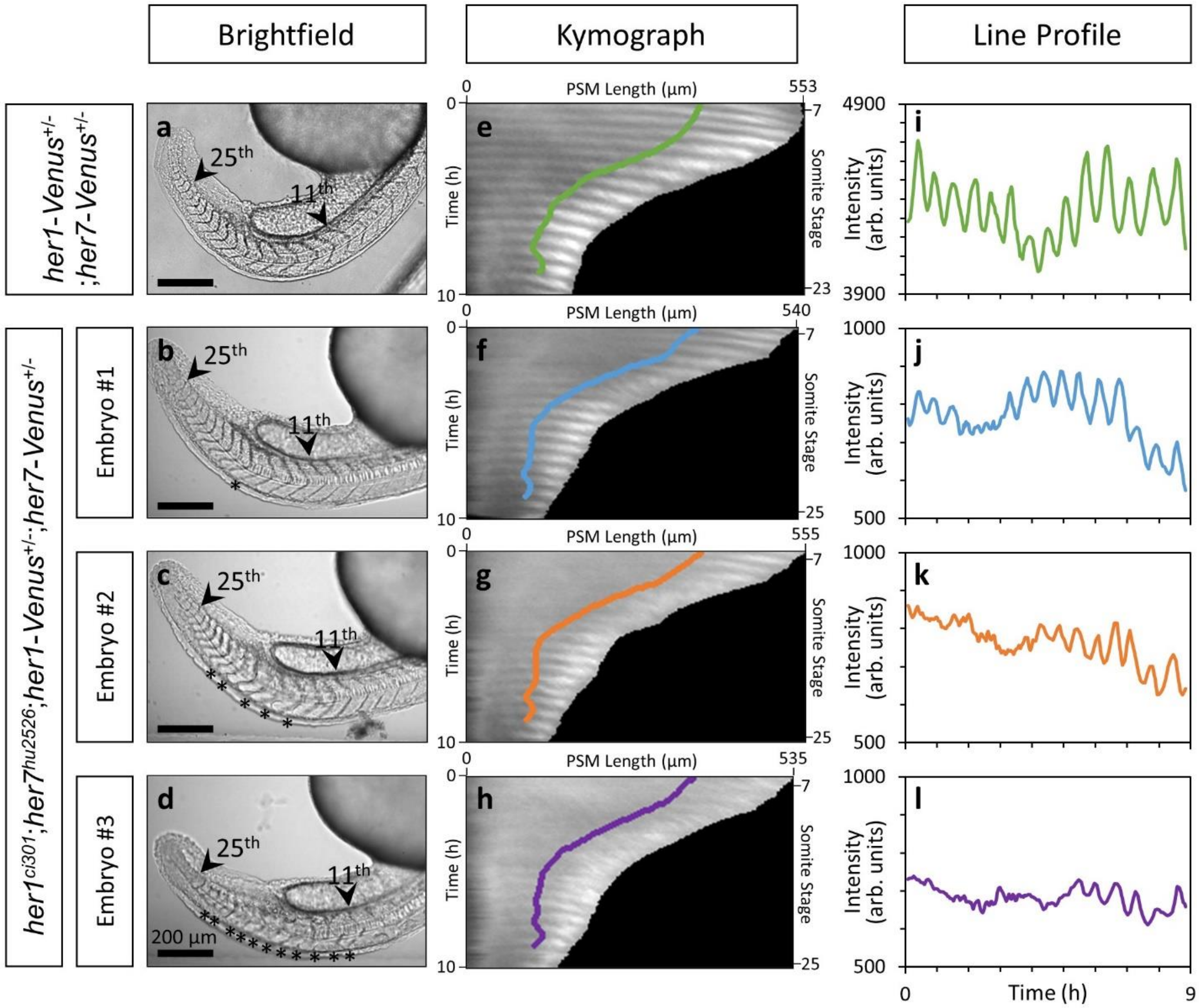

Supplementary Figure 4. Clock expression patterns along the PSM on kymographs and clock amplitudes correlate with phenotypic strengths of wild-type and mutant embryos. **a**-**d** Brightfield images of *her1*-*Venus*$^{+/-}$;*her7*-*Venus*$^{+/-}$ (n = 14, N = 1) (**a**), and *her1*^{ci301};*her7*^{hu2526};*her1*-*Venus*$^{+/-}$;*her7*-*Venus*$^{+/-}$ embryos (n = 41, N = 3) ordered by their increasing number of defective boundaries (**b** through **d**). Stars show defected boundaries between 11th and 25th somite boundaries. Scale bars are 200 µm. **e**-**l** Tail bud aligned kymographs of Venus along the PSM for 10 hours (**e**-**h**), and line profiles (**i**-**l**) along the positions of determination fronts seen on the kymographs for representative embryos, which show wild-type (**e**, **i**, green line), mild (**f**, **j**, blue line), moderate (**g**, **k**, orange line), and strong (**h**, **l**, purple

line) phenotypes. arbitrary units (arb. units). *n* is the number of embryos; *N* is the number of independent experiments.

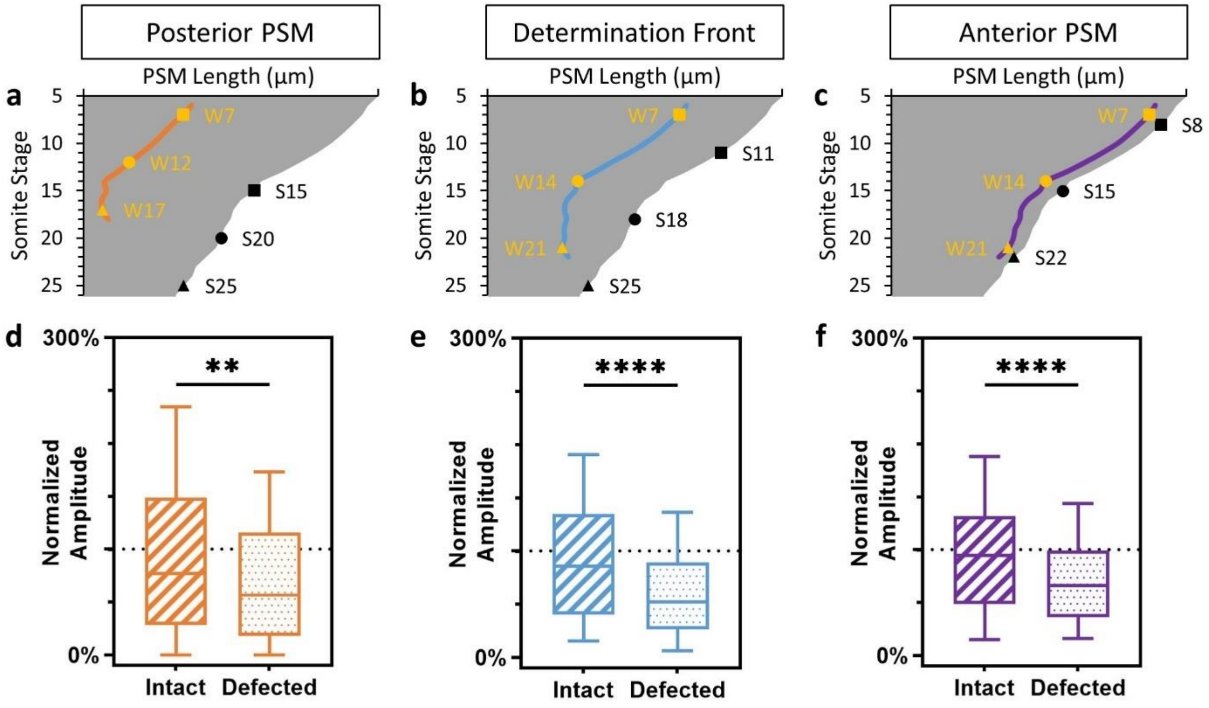

Supplementary Figure 5. The correlations of the clock amplitudes (measured at different locations) with segmentation success. a-c Cartoons show kymographs using PSM lengths between 5 and 26 somite stage as reported in [26]. Amplitudes are calculated along the S-VII (**a**, orange line, posterior PSM), S-III (**b**, blue line, determination front), and S0 positions (**c**, purple line, anterior PSM). **d-f** The amplitudes of oscillations preceding successful segmentation are higher than those preceding failed ones in all three locations, whereas the difference at the determination front is slightly better (median difference: 34.0, n_{intact} = 283, n_{defected} = 328, *N* = 3) than that of anterior PSM (median difference: 28.5, n_{intact} = 248, n_{defected} = 365, *N* = 3), which is better than that of the posterior PSM (median difference: 20.5, n_{intact} = 230, n_{defected} = 214, *N* = 3). ***P* = 0.0080 (posterior), *****P* = 0.4908×10^{-8} (determination front), *****P* = 0.6746×10^{-6} (anterior). The whisker plot shows the median (line), quartiles (box), as well as the 10th and 90th

percentiles (whiskers). *n* is the number of boundaries; *N* is the number of independent experiments.

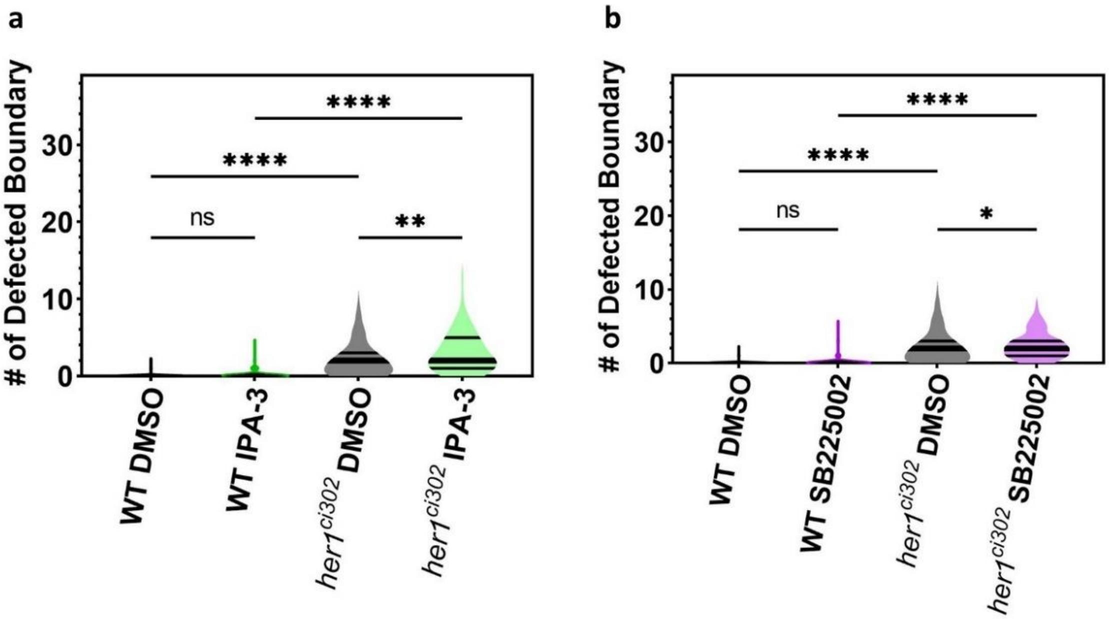

Supplementary Figure 6. The number of segmentation defects increases in a synergistic manner in *her1^{ci302}* mutants. a, **b** Number of defective boundaries per each side of wild-type and *her1^{ci302}* mutants treated with 3 μM DMSO (**a**, **b**, gray) (*n* = 172, *N* = 3, and *n* = 156, *N* = 3), 1 μM IPA-3 (**a**, green) (*n* = 178, *N* = 3, and *n* = 202, *N* = 3), and 1 μM SB225002 (**b**, purple) (*n* = 184, *N* = 3, and *n* = 202, *N* = 3). *P* = 0.9185 (WT DMSO vs WT IPA-3), ****P < 0.0001x10^{-11} (WT DMSO vs *her1^{ci302}* DMSO), ****P < 0.0001x10^{-11} (WT IPA-3 vs *her1^{ci302}* IPA-3), **P = 0.0020 (*her1^{ci302}* DMSO vs *her1^{ci302}* IPA-3), *P* > 0.9999x10^{-11} (WT DMSO vs WT SB225002), ****P < 0.0001x10^{-11} (WT DMSO vs *her1^{ci302}* DMSO), ****P < 0.0001x10^{-11} (WT SB225002 vs *her1^{ci302}* SB225002), *P = 0.0252 (*her1^{ci302}* DMSO vs *her1^{ci302}* SB225002), Kruskal–Wallis ANOVA with Dunn's multiple-comparison correction. ns, not significant. The violin plots show the median (thick black line) and quartiles (thin black lines). *n* is the number of sides; *N* is the number of independent experiments.
