# Supplementary material for: Stochastic gene expression and environmental stressors trigger variable somite segmentation phenotypes
Source: Nat Commun. 2023 Oct 14;14:6497. doi: 10.1038/s41467-023-42220-7 (PMC10576776; doi:10.1038/s41467-023-42220-7)
Supplement: Supplementary file 3 — Reporting Summary [file 41467_2023_42220_MOESM3_ESM.pdf]

nature portfolio

Corresponding author(s): Ertugrul M. Ozbudak

Last updated by author(s): Sep 25, 2023

Reporting Summary

Nature Portfolio wishes to improve the reproducibility of the work that we publish. This form provides structure for consistency and transparency in reporting. For further information on Nature Portfolio policies, see our Editorial Policies and the Editorial Policy Checklist.

Statistics

For all statistical analyses, confirm that the following items are present in the figure legend, table legend, main text, or Methods section.

n/a	Confirmed	
☐	☒	The exact sample size (*n*) for each experimental group/condition, given as a discrete number and unit of measurement
☐	☒	A statement on whether measurements were taken from distinct samples or whether the same sample was measured repeatedly
☐	☒	The statistical test(s) used AND whether they are one- or two-sided<br>*Only common tests should be described solely by name; describe more complex techniques in the Methods section.*
☒	☐	A description of all covariates tested
☐	☒	A description of any assumptions or corrections, such as tests of normality and adjustment for multiple comparisons
☐	☒	A full description of the statistical parameters including central tendency (e.g. means) or other basic estimates (e.g. regression coefficient) AND variation (e.g. standard deviation) or associated estimates of uncertainty (e.g. confidence intervals)
☐	☒	For null hypothesis testing, the test statistic (e.g. *F*, *t*, *r*) with confidence intervals, effect sizes, degrees of freedom and *P* value noted<br>*Give P values as exact values whenever suitable.*
☒	☐	For Bayesian analysis, information on the choice of priors and Markov chain Monte Carlo settings
☒	☐	For hierarchical and complex designs, identification of the appropriate level for tests and full reporting of outcomes
☐	☒	Estimates of effect sizes (e.g. Cohen's *d*, Pearson's *r*), indicating how they were calculated

Our web collection on statistics for biologists contains articles on many of the points above.

Software and code

Policy information about availability of computer code

Data collection	Nikon NIS Elements was used to acquire images at Nikon microscopes.
Data analysis	MATLAB 2022b, GraphPad Prism 9.5.0, Nikon NIS Elements 9.0, Imaris 9.8, Microsoft 365 Excel, and FIJI (ImageJ 1.54f) softwares were used.<br>MATLAB 2022b was used to analyze data from NIS Elements and FIJI. Custom Matlab and FIJI codes are available at GitHub (https://github.com/ozbudak/Keseroglu_2023_Penetrance), which is archived in Zenodo with the identifier (https://doi.org/10.5281/zenodo.8377968).<br>Nikon NIS Elements 9.0, Imaris 9.8, and FIJI (ImageJ 1.54f) were used to analyze live imaging and fixed samples data.<br>GraphPad Prism 9.5.0 and Microsoft 365 Excel were used to plot graphs and statistical analysis.

For manuscripts utilizing custom algorithms or software that are central to the research but not yet described in published literature, software must be made available to editors and reviewers. We strongly encourage code deposition in a community repository (e.g. GitHub). See the Nature Portfolio guidelines for submitting code & software for further information.

Data

Policy information about availability of data

All manuscripts must include a data availability statement. This statement should provide the following information, where applicable:

- Accession codes, unique identifiers, or web links for publicly available datasets
- A description of any restrictions on data availability
- For clinical datasets or third party data, please ensure that the statement adheres to our policy

The original microscopy image files are provided at BioStudies under accession number S-BSST1156 (https://www.ebi.ac.uk/biostudies/studies/S-BSST1156). Source data are provided with this paper. There are no restrictions on data availability. Custom Matlab and FIJI codes are available at GitHub (https://github.com/ozbudak/Keseroglu_2023_Penetrance), which is archived in Zenodo with the identifier (https://doi.org/10.5281/zenodo.8377968).

Research involving human participants, their data, or biological material

Policy information about studies with human participants or human data. See also policy information about sex, gender (identity/presentation), and sexual orientation and race, ethnicity and racism.

Reporting on sex and gender	N/A
Reporting on race, ethnicity, or other socially relevant groupings	N/A
Population characteristics	N/A
Recruitment	N/A
Ethics oversight	N/A

Note that full information on the approval of the study protocol must also be provided in the manuscript.

Field-specific reporting

Please select the one below that is the best fit for your research. If you are not sure, read the appropriate sections before making your selection.

☒ Life sciences ☐ Behavioural & social sciences ☐ Ecological, evolutionary & environmental sciences

For a reference copy of the document with all sections, see nature.com/documents/nr-reporting-summary-flat.pdf

Life sciences study design

All studies must disclose on these points even when the disclosure is negative.

Sample size	No statistical methods were used to predetermine sample size, but the sample sizes are similar to previous studies (Choorapoikayil, S., et al. PLoS One 7(6) 2012; Richter, S., et al. Nat Commun 8, 1901, 2017; Zinani, O.Q.H., et al. Nature 589, 431–436, 2021). Sample sizes are feasible number of embryos harvested from multiple (at least 3 pairs of) mated parents within a day that can later be mounted and imaged within a reasonable duration of experiments. Each independent experiment are further repeated at least twice as indicated throughout the manuscript.
Data exclusions	If a tissue is severely cut or bent during mounting, we excluded it from further analysis.
Replication	Embryos were collected from at least 3 pairs of fish breedings. Data come from at least two independent experiments. All replication experiments gave similar results. All samples were pulled together during analysis.
Randomization	Not applicable for our study. Samples were separated based on genotype, environmental conditions or drugs treated only. Embryos were always used from petri dish mixture of multiple clutches, automatically randomized.
Blinding	Blinding was mostly not possible. Phenotypes of boundaries were discern by at least two experimentalists, and the data from different batches were obtained and analyzed separately by two experimentalists for all experiments. Analysis was confirmed by at least three authors of the manuscript.

Reporting for specific materials, systems and methods

We require information from authors about some types of materials, experimental systems and methods used in many studies. Here, indicate whether each material, system or method listed is relevant to your study. If you are not sure if a list item applies to your research, read the appropriate section before selecting a response.

Materials & experimental systems

n/a	Involved in the study	
☐	☒	Antibodies
☒	☐	Eukaryotic cell lines
☒	☐	Palaeontology and archaeology
☐	☒	Animals and other organisms
☒	☐	Clinical data
☒	☐	Dual use research of concern
☒	☐	Plants

Methods

n/a	Involved in the study	
☒	☐	ChIP-seq
☒	☐	Flow cytometry
☒	☐	MRI-based neuroimaging

Antibodies

Antibodies used	Chicken monoclonal IgY anti-GFP (Cat# A10262, Invitrogen, Lot# 2321831, 1:200) and rabbit polyclonal Living Colors anti-DsRed (mCherry; Cat# 632496, Takara, Lot# 2103116, 1:200) were used as primary antibodies. Alexa Fluor 488 goat anti-chicken IgG H+L (Cat# A11039, Invitrogen, Lot# 2420700, 1:200) and Alexa Fluor 594 donkey anti-rabbit IgG H+L (Cat# A21207, Invitrogen, Lot# 2066086, 1:200) were used as secondary antibodies.
Validation	Antibodies were validated by the manufacturer-information obtained from the manufacturers' websites. For chicken monoclonal IgY anti-GFP (Cat# A10262, Invitrogen, Lot# 2321831): https://www.thermofisher.com/antibody/product/GFP-Antibody-Polyclonal/A10262. For rabbit polyclonal Living Colors anti-DsRed (mCherry; Cat# 632496, Takara, Lot# 2103116): https://www.takarabio.com/documents/Certificate%20of%20Analysis/632496/632496-101717.pdf. Antibodies were used against membrane-GFP or membrane-Cherry, respectively, to detect cell borders during cell segmentation analysis of RNA injected embryos. The antibodies did not demonstrate any signal in uninjected controls, validating their function.

Animals and other research organisms

Policy information about studies involving animals; ARRIVE guidelines recommended for reporting animal research, and Sex and Gender in Research

Laboratory animals	Tg(her7:her7-Venus)ci303, Tg(her1:her1-Venus)bk15, her1ci302, her7hu2526, her1ci301;her7hu2526, and AB wild-type adult fish were used in experiments. Adult fish between 8 and 18 months old are used in matings.
Wild animals	None
Reporting on sex	Sex is not chromosomally determined in zebrafish. We used up to 2 days old embryos. Sex is not determined at this stage. Thus, we did not discriminate on a particular sex in our studies.
Field-collected samples	None
Ethics oversight	All of the fish experiments were performed under the ethical guideline of Cincinnati Children's Hospital Medical Center, and the animal protocol was reviewed and approved by Cincinnati Children's Hospital Medical Center Animal Care and Use Committees (Protocol #2020-0031).

Note that full information on the approval of the study protocol must also be provided in the manuscript.
